# The Knowledge Translation Pizza-Dilemma: A Response to Recent Commentaries

**DOI:** 10.34172/ijhpm.2023.8296

**Published:** 2023-10-18

**Authors:** Robert A.J. Borst, Rik Wehrens, Roland Bal

**Affiliations:** Erasmus School of Health Policy & Management, Erasmus University Rotterdam, Rotterdam, The Netherlands.

*Rules of Taste Enforce Structures of Power*.

**Susan Sontag**^1^

 There is a special place on the internet, which is devoted to pizza metaphors and quotes. Somehow, the seven insightful commentaries^2–8^ on our paper remind us of that place – in a good way. We thank the authors of these commentaries for investing their time and efforts in writing such eloquent and often generous reflections on our review that was published in this Journal.^9^ In this response we seek to draw out potential cross-pollinations, but also address nuances that we feel might not have come across so clearly in the original paper. To set the stage for our response, we will first return to our pizza metaphor.

 Generally speaking, most people know what ‘a pizza’ looks like – that is: an oven-baked, relatively flat piece of dough that usually resembles a circular shape. Yet when you ask different people what constitutes a ‘good’ pizza, the answers are less univocal: pizzas need to have a thick crust, a thin crust, must be cheesy, or not, definitely do not have pineapple on them, or might include pineapple as a mere guilty pleasure after all. Some people may dislike pizzas entirely. We believe that it is not far-fetched to extend this to the past and current debates on our ‘pizza,’ namely: knowledge translation (KT). There is recurring sentiment in the literature which suggests that KT is one ‘thing’ – a thing that can be tinkered with, and which can be attuned to the preferences of its practitioner or scholar; much like the dough and toppings of a pizza. Most KT scholars and practitioners will have a preferred ‘flavour’ of KT and some may discredit anyone who dares to add ‘pineapple’ to their flavour.

 In the pizza-like understandings of KT, there are core ingredients of KT. Yet while reading some commentaries on our paper, we could not help but wonder: are we understanding ‘KT’ in a comparable way after all? Could it be that our flavour of KT is not a pizza at all? Ødemark,^6^ for instance, argues that we are prescribing a sequential form of KT – which we certainly did not intend to. Interestingly, Sturmberg^8^ relates our thinking to the notion of ‘complex adaptive organisations,’ even though this is not the area that our paper addresses. Our pizza might simply have too many toppings (which some people, arguably, also prefer) and starts to resemble a casserole. In the likely event that this represents our failure to express our position on KT in a clear and concise way, we will briefly expand on what we see as KT and how that affects our suggestion to study practices of (sustaining work in) KT.

 In our work, we see KT as a descriptor of a loosely demarcated phenomenon whereby actors in various ways, and through different means, seek to build relations and relay knowledge into different practices with the overall aim of affecting those practices. Such a perspective, as Oliver^7^ argues, liberates KT from its linear shackles, for instance by not merely stating that relations are important, but by showing what it actually means to build such relations in KT work. Inspired by the commentaries, we feel that it is important to tease out two more contributions to the KT literature here. First, when we speak of ‘translation’ in our review, that does not include any presumptions about directionality or gradience, yet it does signify a displacement– when knowledge is translated, it does not stay at the same place. Ødemark’s^6^ argument that the ‘receiving end’ is not an empty void, in need of enlightenment, is of crucial importance here. Hence translation connotes more a process of weaving things together, or relaying (cf. Haraway^10^), than of transmitting. Beside displacement, translation also implies transformation: by being translated, knowledge – which necessarily includes embodied, tacit, experiential, *and* scientific knowledge – does not stay the same in form and nature.^11,12^ Instead, it becomes part of a new network, which requires the creation of new connections that were not there before – such work indeed *also *needs to be done alongside the *“production and dissemination of new knowledge in ways that enhance its utility to end-users.”*^2^ This is particularly important considering that translations are never neutral and have the potential to affect the lives and well-being of citizens worldwide.^4^

 Second, when we speak of practices, we do not necessarily refer to the commonly use triad of ‘research, policy, and practice’. ‘Practice,’ in our understanding, signifies actors who are practising something, which might include situated actions in (health)care, research, or policy. It is about studying activities of actors and how they transpire in certain processes. Above all, what counts as KT is empirically defined as any activity related to the translation of knowledge. Depending on where such practices are studied, this means that KT may now comprise much more, including very mundane interactions, and not only the application of a set of tools specifically designed for KT.^13,14^ Finally, and to be clear: in our understanding KT is certainly not confined to clinical settings at all, although we do acknowledge that this is what has historically and – to our opinion – problematically imprinted much of the field. We therefore enthusiastically welcome the plea of Edelman and Topp^2^ to extend our thinking to ‘non-clinical settings’ and that such populations also necessitate different forms of KT. Kothari and Cameron^3^ eloquently capture that this also includes *“rebalance[ ing ] the power of underserved voices or hidden knowledge through KT work”* – they are absolutely right in noting that any process of doing and sustaining KT does not solely rely on researchers or explicit ‘knowledge producers,’ but revolves in the wider constellation of caretakers, community members, or other – often underserved – voices. Following how KT travels and is provided meaning locally thus forms an important area of future study (cf. Abrahamsson and Mol^15^).

 Having laid out our perspective on KT, we feel that it appropriate to briefly state here that we ourselves are not KT practitioners (at least not in the strict sense). We do, however, study different *practices* of KT. Similarly, our analyses do not aim to provide a prescriptive model for how KT ought to be done, but a description of how KT is practised and what we might learn from that. It would, however, be dishonest to argue that our conclusions are entirely harmless and do not include, albeit implicit, a plea for practising KT in a different way. In line with Meier,^5^ we feel that it is time to empirically open-up KT (cf. Borst et al^14^) and expand it beyond ‘common-taste’ KT (our words). Such expansions and extensions also allow for understanding the role of contestation in processes of KT – not merely about the tools and instruments that are used, but also about what is, and is not, considered as valid knowledge. Finally, we wholeheartedly welcome Oliver’s^7^ plea to study and describe *“what it takes” *to do certain KT work, rather than staying with narrow frameworks and indicator sets. Instead, we may find inspiration in empirically disentangling more undervalued aspects of KT – which may appear boring, but actually provide insight into crucial underlying mundane work.

## Acknowledgements

 The authors would like to thank Alexandra Edelman, Stephanie Topp, Anita Kothari, Jacqui Cameron, Tanja Kuchenmüller, Laura dos Santos Boeira, Ninna Meier, John Ødemark, Kathryn Oliver, and Joachim Sturmberg for writing such stimulating commentaries – we will happily share a pizza with them should that occasion arise.

## Ethical issues

 Not applicable.

## Competing interests

 Authors declare that they have no competing interests.
